# Small temperature differences can improve the performance of mesophilic sludge-based digesters

**DOI:** 10.1007/s10529-017-2418-y

**Published:** 2017-08-28

**Authors:** Maja Nielsen, Christian Holst-Fischer, Bjørn Malmgren-Hansen, Michael Bjerg-Nielsen, Caroline Kragelund, Henrik Bjarne Møller, Lars Ditlev Mørck Ottosen

**Affiliations:** 1 Department of Engineering, Aarhus University, Hangøvej 2, 8200 Aarhus N, Denmark; 2 Danish Technological Institute, Teknologiparken, Kongsvang Allé 29, 8000 Aarhus C, Denmark

**Keywords:** Anaerobic digestion, Full-scale plants, Mesophilic operation, Methane yield, Sludge digestion

## Abstract

**Objective:**

To assess the effect of small temperature increases in mesophilic sludge-based digesters in order to develop and evaluate strategies for improving the biogas production in full-scale digesters.

**Results:**

Methane production was strongly affected by small temperature differences, and this result was consistent across samples from 15 full-scale digesters. The specific methane yield varied between 42 and 97.5 ml g VS^−1^ after 15 days of incubation at 35 °C, and improved when increasing the digester temperature to 39 °C. Only a limited quantity of additional gas was required to balance out the cost of heating and a positive energy balance was obtained. Further increases in temperature, in some cases, negatively affected the production when operated at 42 °C compared to 39 °C.

**Conclusions:**

Small temperature increases should be applied to mesophilic sludge-based digesters to optimize the biogas production and is applicable to digesters operated in the lower mesophilic temperature range.

## Introduction

Technologies producing renewable energy have gained more attention as the interest in phasing out fossil fuels has increased. Anaerobic digestion (AD) is one of the most promising technologies, and it is already applied at full-scale around the world. This microbial mediated process catalyzes the degradation of a variety of wastes, facilitating the production of biogas containing the energy-carrier methane (Mata-Alvarez et al. 2014; Weiland 2010). The produced methane is an important resource for gas, electricity, heat and fuel, serving as an important substitute to fossil fuels and achieving a more sustainable energy production.

Biogas systems are widely used in the disposal of agricultural wastes and are also commonly implemented at wastewater treatment plants (WWTPs), originally for the stabilization of putrescible solids in Denmark and other European countries (Gonzalez-Gil et al. 2016; Kelessidis and Stasinakis 2012). However, there has been an increasing interest in applying the AD-technology to the production of renewable energy (Deublein and Steinhauser 2011). This requires a thorough knowledge of the AD-process, and an identification of the most energy-efficient optimization strategies for sustainable reactor operation.

The factors affecting reactor performance can be divided into three classes: (i) feedstock characteristics, (ii) reactor design, and (iii) operational conditions (Cioabla et al. 2012). Understanding the interactions between the operational parameters and the microbial communities is essential in the AD-operation. Temperature is one of the most essential parameters in AD and, in most cases, correlates the methane production (Chapleur et al. 2016; Kim and Lee 2016). The reaction velocity, the dominance of certain biochemical pathways, and microbial activity are some of the areas known to be affected by temperature (Appels et al. 2008). Hence, paying attention to the reactor temperature is essential, since minor temperature differences can significantly affect the reactor performance and the methane yield. Most often, experimental-validated results are not used to select the specific operational temperature, e.g., within the mesophilic temperature range. Further analysis of the effect of minor temperature transitions could result in the development of new and more energy-efficient optimization strategies.

AD is usually operated either at psychrophilic, mesophilic or thermophilic conditions (Jain et al. 2015). Thermophilic operation, as a rule, results in higher yields compared to mesophilic operation; thus, temperature transition has been used as one strategy to optimize the reactor performance (De Vrieze et al. 2016; Moset et al. 2015). However, the success of temperature transition and thermophilic operation may depend on a balanced interplay across the microbial communities. Thus, the capability to adapt to new operational conditions is essential (Westerholm et al. 2017). More unstable reactor performance has also been reported at thermophilic conditions, which reflects the downside of operating in this temperature range (Labatut et al. 2014). Thermophilic operation also requires additional energy-input compared to mesophilic operation. The increased biogas production and heating requirements need to balance each other out for a positive net-energy yield (Ge et al. 2011). Therefore, this implies that there should be a larger focus on the effect of minor temperature differences in AD; for instance, in mesophilic conditions. Thereby, a balance between the capital expenditure and the operation and maintenance expenditure can be achieved (Hadidi and Omer 2017).

Treatment efficiency of primary and excess sludge by means of AD is highly dependent on the hydrolysis step, which is considered rate-limiting (Appels et al. 2008). Thus, improving the hydrolysis rate could significantly increase the reactor performance and the biogas yield (Carrere et al. 2016). Different pretreatment strategies (e.g., mechanical, enzymatic, or thermal hydrolysis) facilitate access to the consumable compounds and improve the AD-process (Wahid et al. 2015). However, one of the downsides is the significant investment costs and the additional energy required for the operation of these technologies, pointing in the direction of finding alternative optimization strategies.

The objective of the present study was to identify strategies to optimize mesophilic biogas production. Fifteen full-scale digesters were sampled, and the residual methane yield was determined in batch-incubations at three different temperatures (35, 39, and 42 °C). The energy balances were calculated for each scenario to evaluate the energy efficiency of the operational modifications. Thereby, this study fills a significant gap in the literature and provides the evidence of the importance and feasibility of minor temperature differences in biogas production.

## Materials and methods

### Sample and data collection

Fifteen anaerobic digesters (ADs) from 12 wastewater treatment plants (WWTPs) were sampled. All plants are located in Denmark. The reactors were sampled from October 2014 to September 2015. The ADs were all continuously stirred tank reactors (CSTRs) that operated at mesophilic conditions. All digesters had been running for more than 1 year and showed normal operating conditions prior to sampling. The reactors were coded as follows: all of the plants sampled were marked with a letter from A to M and followed by a number referring to the times of sampling. Two parallel reactors were sampled at two of the WWTPs included in the study. In such cases, the second number in the sample ID refers to the number of the reactor sampled at the plant. The samples were stored at 4 °C and shipped to the laboratory within 24 h after sampling. The experiment was conducted at a maximum of 48 h after sampling. The samples for the chemical analyzes were examined immediately upon arrival at the laboratory. The operational parameters were reported by the operators at the AD installations.

### Impact of temperature at methane production

The automatic methane potential test system (AMPTS) II (Bioprocess Control, Sweden AB) was used to evaluate the impact of minor temperature differences. The system consists of 15,650 ml glass bottles. 400 ml digester material was transferred to replicate reactors and incubated at 35, 39 or 42 °C. All sampled full-scale digesters were operated within this temperature range. Temperatures were monitored on a daily basis. Replicate bottles were prepared for each of the examined temperatures, and the bottles were not transferred from one incubation-temperature to another. Additional compounds were not added to any of the batch-reactors. Each of the reactors was stirred in cycles of 60 s followed by a 60 s pause. CO_2_ was precipitated in NaOH, and only methane was detected in the flowmeter. The incubations were run for 30 days and the impact of temperature on the methane yield (ml g VS^−1^) was evaluated. The methane yield was calculated based on the volatile solids (VS) content, and the values in the initial samples were used in the analysis. The results from day 5, 10, and 15 were chosen for further analysis to best simulate continuous reactor conditions.

### Energy balance

To determine the feasibility of the temperature modifications, the energy balances were calculated. The additional gas and energy needed for heating and maintaining temperature were included in the calculation, applying a heat capacity of sewage sludge of 1.16 kWh tonnes^−1^ K^−1^ (Møller et al. 2008). The average temperature of the incoming sludge was estimated as 8 °C, and the expected heat loss (HL) from the digester surface was calculated with the equation:

$$ HL = A*K* \Delta T, $$

where A is the surface area for the digester and K is the heat loss from the surface, which is set at 0.2 J m^−2^ K^−1^ and equals to approximately 200 mm insulation. Δ*T* is the average difference between the temperature inside the digester and the surrounding temperature, which for Danish conditions is approx. 8 °C on average.

The heat loss was calculated with digesters at 35, 39, 42 °C and a 4000 m^3^ cylindrical 12 m high digester. In the calculation, a hydraulic retention time of 20 days was assumed.

### Physicochemical analysis

The chemical composition of the digester material was determined according to standard procedures. The digistate from the full-scale digesters were analyzed immediately after arrival to the laboratory. The batch-sludge was not analyzed after the batch test. To determine the content of total solids (TS) and volatile solids (VS), the samples were dried at 105 °C and burned at 550 °C (American Public Health Association (APHA) 2005). NH_4_^+^ was measured spectrophotometrically using a commercial kit. Fat was determined according to the Schmid-Bondzynski-Ratzlaff method (ISO^2004^), and the lignin content was measured according to Van Soest et al. (1991). Dissolved volatile fatty acids (VFA) were separated using a GC equipped with a flame ionization detector. A HP-INNOWAY column (30 m, 0.250 mm, 0.25 µm) (Agilent Technologies) was used with (He) as carrier gas.

## Results

### Digester sample characterization

Twelve full-scale AD installations, including 15 digesters, were sampled. The digester operators reported stable digester performance prior to the time of sampling. Hence, the results in the present study were not assumed to be biased by unstable performance. However, some differences were observed in the operational parameters controlled by the operators. The content of VFA, for instance, varied between 31.8 and 504 mg l^−1^ and the HRT varied between 20 and 40 days (Table 1). The ADs were operated in the mesophilic range 33–41 °C. The chemical composition of the digester samples was analyzed (Table 2) and the content of TS varied between 1.67 and 3.62%. VS were 39.3–64.1% of TS. The content of protein varied between 2 and 45.4% TS and the quantity of carbohydrates was between 5.8 and 40% TS, except for one outlier. Lignin was 7.8–17.7% TS and fat constituted up to 6.1% TS. Fats were detected in only six of the analyzed digesters. The percentage of TS degraded (after 30 days) was 5.9–13.3%, determined on the basis of five samples (data not shown). The pH was between 7.3 and 7.9 (Table 1) and the content of NH_4_^+^ varied between 450 and 1040 mg l^−1^ (data not shown).

**Table 1:** Overview of the operational parameters in the sampled full-scale digesters

Plant	Capacity (m^3^)	Biogas (m^3^ day^−1^)	[CH_4_] (%)	HRT (days)	Temperature (°C)	pH (–)	VFA (total) (mg l^−1^)
A	2400	–	63	35–40	38	7.65	31.8
B	810	1081	62	22	39.7	7.91	34.8
C	990	550	65	23.8	35	7.51	49.4
D.1	1600	1882	62.5	27	37.8	7.49	192
E	2800	8518	60.9	28	38.2	7.57	498
F	1000	3350	60	22–25	35	7.27	504
G	600	120	65	21	36.4	7.87	324
H	700	–	63	35	33.5	7.9	373
D.2.1	1600	1883	62.5	27	36.8	7.52	246
D.2.2	1600	1883	62.5	27	37.4	7.43	324
I.1.1	2600	1920	64–65	20–22	37	7.63	451
I.1.2	2600	1920	64–65	20–22	34.5	7.66	435
K	1765	1600	60–65	20–24	33	7.5	54.1
L	2000	–	65	25–30	37	7.49	45.6
M	2200	1200	63	35	38.1	7.38	38.9

Reactor capacity (m^3^), daily biogas-production (m^3^ day^−1^), percentage of methane in the biogas (%), hydraulic retention time (days), temperature (°C), pH and volatile fatty acids (mg l^−1^). The digester temperature was the average temperature 1 month prior to the date of sampling

**Table 2:** Chemical composition of the full-scale digester samples

Plant	Temperature^a^ (°C)	TS (%)	VS (%TS)	Inorganic (%)	Protein (%TS)	Other carbohydrates (%TS)	Lignin (%TS)	Fat (%TS)
A	38	3.17	58.5	41.5	2.46	39.5	16.6	nd
B	39.7	3.35	57.5	42.5	2	40	15.5	nd
C	35	2.72	57.5	42.5	45.4	0	14.1	nd
D.1	37.8	1.95	63.2	36.9	7.05	32.7	17.7	5.6
E	38.2	3.57	61.6	38.5	18.5	28.3	14.8	nd
F	35	3.60	62.1	37.9	21.3	23.1	13.8	3.9
G	36.4	3.19	39.3	60.7	5.48	25.6	8.17	nd
H	33.5	1.75	59.1	40.9	12.5	38.9	7.78	nd
D.2.1	36.8	1.86	59	41	12.5	29.6	16.9	nd
D.2.2	37.4	1.67	59.1	40.9	13.1	29.6	16.4	nd
I.1.1	37	2.70	61.6	38.4	15.2	34.7	11.7	nd
I.1.2	34.5	3.12	61.1	38.9	13.1	32.1	12.7	3.2
K	33	3.62	63	37	16	29.7	13.1	4.3
L	37	3.19	60.8	39.2	16.8	25.6	12.3	6.1
M	38.1	2.36	64.1	35.9	36.4	5.8	17.2	4.7

The specific procedures for the individual analysis are presented in the main text

^a^Refers to the average temperature in the sampled digesters 1 month prior to sampling

*nd* not detected

### Effect of temperature on the methane production

**Fig. 1:**
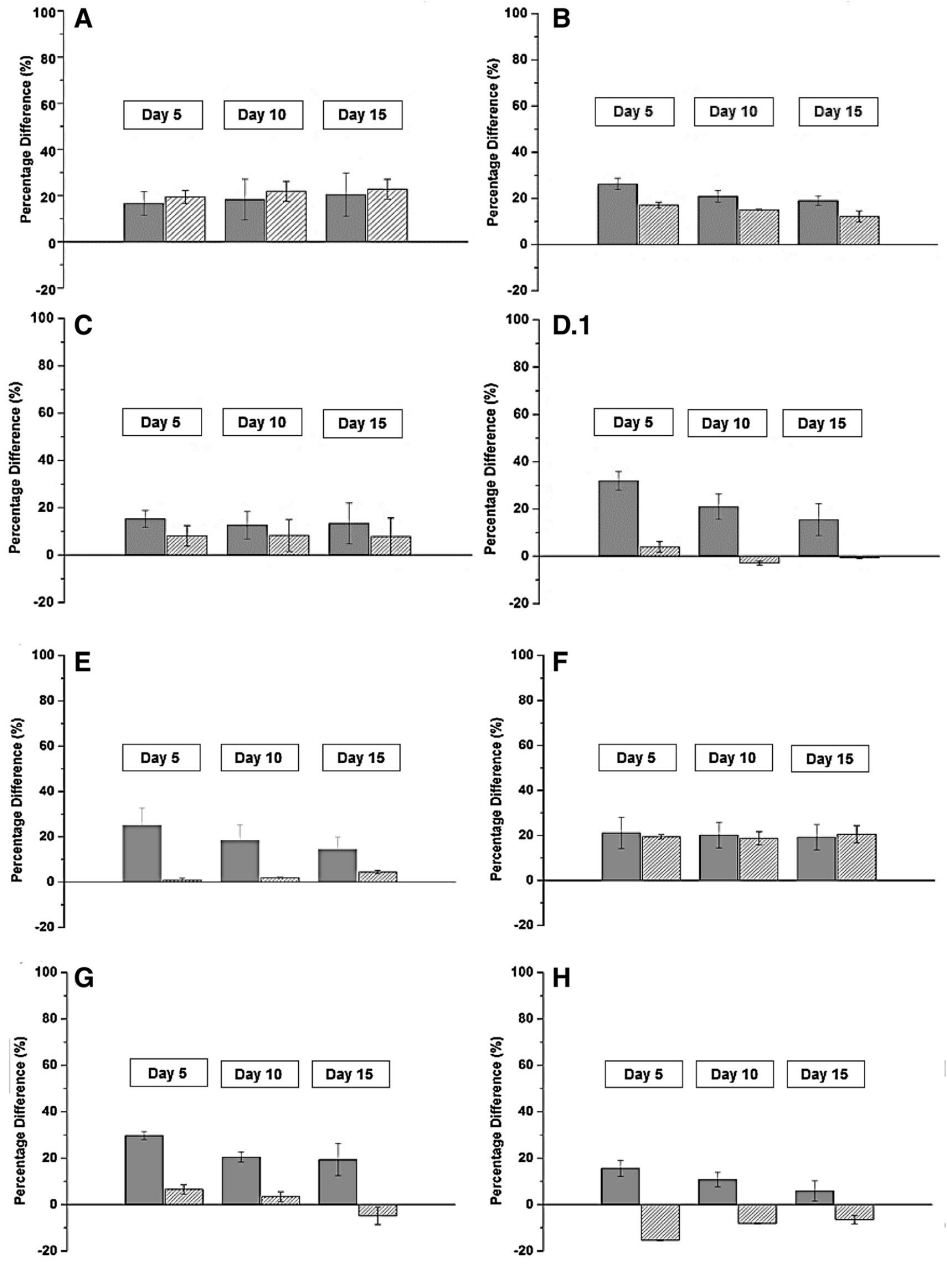

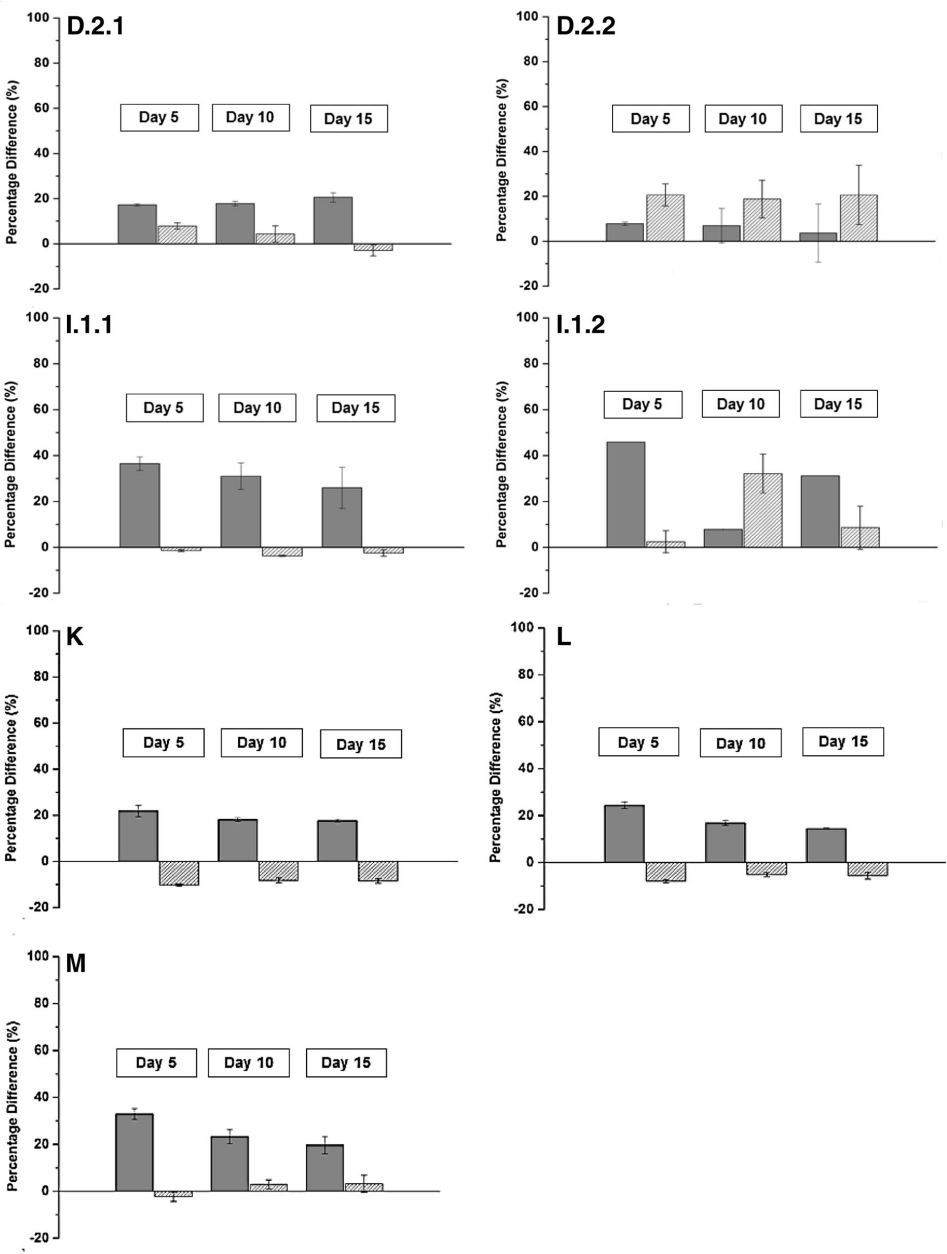
Percentage differences (%) of the specific methane yield (ml CH_4_ g VS^−1^) in incubation at 39 °C relative to 35 °C (*gray*) as well as 42 °C relative to 39 °C (*white, dense-striped*). The results from day 5, 10, and 15 are presented. Standard deviations are shown for duplicate reactors. The plant ID is indicated in the *upper left corner* and corresponds to the annotation used elsewhere in the study

methane production was examined at three temperatures. An increase in temperature correlated positively with the methane yield (Fig. 1). The effect was most pronounced in the lower range of the tested temperatures (the difference between samples incubated at 35 and 39 °C), although an increase, however minor, was also observed in the batch incubations operated at 42 °C. However, in some cases, e.g., plant H, methane production was inhibited in the upper temperature range. The specific methane yield varied between 28 ± 0.61 and 84.2 ± 0.25 ml g VS^−1^ (day 5), 36.9 ± 0.71 to 99.5 ± 1.18 ml CH_4_ g VS^−1^ (day 10) and 42.1 ± 0.69 to 106.5 ± 2.61 ml CH_4_ g VS^−1^ (day 15) across all samples (Table 3). Only the results from the batch-reactors operated at 35 °C are depicted in Table 3. The results corresponded to production rates between 5.6 ± 0.12 and 16.8 ± 0.05 ml CH_4_ g VS^−1^ day^−1^ (day 5), 3.69 ± 0.07–9.95 ± 0.12 ml CH_4_ g VS^−1^ day^−1^ (day 10), and 2.81 ± 0.05–7.1 ± 0.17 ml CH_4_ g VS^−1^ day^−1^ (day 15).

**Table 3:** Specific methane yield (ml CH_4_ g VS^−1^) determined at day 5, 10 and 15 presented as well as the corresponding full-scale digester ID for the incubations at 35 °C

Plant	Temperature^a^ (°C)	Specific methane yield (ml CH_4_ g VS^−1^)		
		35 °C, day 5	35 °C, day 10	35 °C, day 15
A	38	32 (±0.81)	39.4 (±2.01)	42.6 (±2.26)
B	39.7	28 (±0.61)	36.9 (±0.72)	42.1 (±0.69)
C	35	48.3 (±0.11)	58.4 (±0.2)	62.7 (±1.35)
D.1	37.8	56.7 (±0.39)	72.6 (±2.63)	81.8 (±5.33)
E	38.2	48.4 (±1.51)	62.2 (±1.40)	69.9 (±1.05)
F	35	38.7 (±1.75)	50.3 (±1.75)	57.3 (±1.75)
G	36.4	37.8 (±0)	47.7 (±0)	53.1 (±0)
H	33.5	64.4 (±1.76)	84.7 (±3.53)	97.5 (±5.5)
D.2.1	36.8	48.8 (±0.22)	56.7 (±0.17)	60.6 (±1.64)
D.2.2	37.4	54.4 (±1.93)	64.7 (±1.41)	71.3 (±3.01)
I.1.1	37	44.8 (±0)	62.5 (±0)	73.7 (±0)
I.1.2	34.5	42.5 (±0)	56 (±0)	64.5 (±0)
K	33	48.8 (±0.29)	61.7 (±0.9)	67.6 (±1.6)
L	37	67.6 (±0.97)	85.1 (±2.03)	93 (±2.19)
M	38.1	45.6 (±0.83)	62.6 (±0.73)	71.9 (±0.54)

The standard deviations from duplicate reactors are shown in the brackets

^a^Refers to the average temperature in the sampled digesters 1 month prior to sampling

The effect of temperature was evaluated in greater detail by comparing the yield obtained at 39 °C relative to 35 °C (lower range) and similarly for 42 °C relative to 39 °C (upper range) (Fig. 1). In this way, the dependence of temperature on the methane yield was identified. The methane yield and temperature in the lower temperature area correlated positively in all samples. The increase varied between 4.27 ± 0.25 and 19.5 ± 0 ml CH_4_ gVS^−1^, 4.4 ± 0 to 19.4 ± 3.58 ml CH_4_ g VS^−1^, and 2.44 ± 9.14 to 20.15 ± 0 ml CH_4_ g VS^−1^ after 5, 10 and 15 days of incubation, respectively (data not shown). This corresponded to a percentage increase between 7.85 ± 0.73 and 45.9 ± 0%, 7.86 ± 0 to 31.1 ± 5.74%, and 3.7 ± 13 to 31.3 ± 0% at 39 °C relative to 35 °C after 5, 10, and 15 days of incubation, respectively (Fig. 1). Thus, the difference remained more or less constant during the time of incubation.

Whether a similar pattern could be observed by further increasing the temperature was examined: the difference between yields at 39 and 42 °C was evaluated. Further increase in temperature was less effective in the upper temperature range. The percentage difference ranged between −15.3 ± 0.2 and 20.7 ± 4.96%, −8.08 ± 0.18 and 32.2 ± 8.47%, and −8.47 ± 1.01 and 20.7 ± 13.21% after 5, 10 and 15 days incubation, respectively (Fig. 1).

### Energy balances comparing cost and profit

Extra energy to heat the reactors is needed to increase the operational temperature. The energetic cost of heating needs to be exceeded by the energy from the extra quantity of the produced gas to achieve a positive energy balance. Of the required energy, most will be used for heating the sludge, whereas the heat loss through insulation is almost negligible. The calculation of the energy balances showed that an additional 0.46 m^3^ CH_4_ tonnes^−1^ sludge was required to offset the additional costs of operation created by changing from 35 to 39 °C. By further increasing the temperature from 39 to 42 °C, 0.35 m^3^ CH_4_ tonnes^−1^ sludge was required to cover the energy cost of increasing the operating temperature. If the original incoming raw-sludge was assumed to produce 0.2 m^3^ CH_4_ kg VS^−1^ and set to contain 4% TS with 80% as VS, this corresponded with the need for an additional 6.3 and 4.2% of gas. The calculations above were made for AD systems not applying heat recovery of the sludge leaving the digester. However, if the heat from the degassed material is re-used, only a very limited quantity of extra gas is required to reach a positive energy balance, and less than 0.1% extra methane is needed. This additional measure would transform the minor increase in the operational temperature into an even more feasible strategy to improve the reactor performance.

## Discussion

Though it has been studied for decades, the optimization of biogas production is still a major focus area in AD-related research. A variety of technologies exist for substrate pretreatment to make recalcitrant compounds more accessible to the microbial communities and the production of biogas. Temperature is one of the key operational parameters in regulating the reactor performance. However, the requirement of additional energy input makes it difficult to obtain a positive energy balance (Wahid et al. 2015) and the effect of minor temperature differences as an operational strategy has almost been completely overlooked. The present study, therefore, fills a significant gap in the literature. The significance of this research lies in its identification of energy efficient strategies to optimize mesophilic biogas production. In the present study, temperature related positively to the methane production, and this trend was particularly clear during the initial days of incubation (Fig. 1). The largest difference was observed between samples incubated at 35 and 39 °C, but only a minor effect was observed by further increasing the temperature to 42 °C. Similar results have been observed elsewhere, e.g., Boušková et al. (2005) and in Kim and Lee (2016), indicating an imbalance in the microbial AD-network and reduced activity at the upper mesophilic temperature range. Hence, the modification of the reactor temperature is most beneficial only in full-scale digesters operated at the lower mesophilic temperature range. No clear pattern was observed regarding the initial temperature in the full-scale digesters and the percentage effect of the temperature differences. This indicates that the temperature in the sampled full-scale digester does not play a role in the response to the induced temperature differences in the batch-reactors. The results from the physicochemical analyses were all within an acceptable range for normal operation. Hence, VFAs were not considered to be the inhibiting factor of methane production. Since very diverse VFA-concentrations were measured in the full-scale digesters, the responses by the microbial communities would also be different, since being pre-adapted to different levels of the fermentation products.

As reactor performance responded immediately to temperature changes, it seemed that the cell-specific activity was the key-factor affecting this performance, and the relative abundance was assumed to be a less important factor. Instead, functional redundancy and the functional potential of individual populations may play a more significant role (Vanwonterghem et al. 2016). Some of the full-scale digesters sampled for this study were also fed with industrial wastes, which may have selected for microbial communities that are specialized to consume different substrates (Sundberg et al. 2013). This supports the idea that functional redundancy is essential and being more important than the actual diversity in the community structure (Briones and Raskin 2003).

The calculations of the energy balances, showed the potential of the additional energy input to be balanced by the extra quantity of produced gas. The results presented here are relevant for several types of biogas plants, e.g., smaller plants that do not have resources for new investments such as heat-exchangers. Since the present study was performed at batch-conditions, future research should test the effect of small temperature modifications in continuously operated digesters. This may provide important insights into the community composition and the functional redundancy, leading to the design of rational engineered energy-technologies.

## Conclusion

Minor temperature modifications are a feasible strategy to optimize the production of biogas. By increasing the reactor temperature from 35 to 39 °C, approx. 20 ml CH_4_ g VS^−1^ extra gas was gained in the batch-reactors. This serves as an important alternative to common pretreatment strategies, e.g., pressure-cooking, extrusion, and methanization, because of the minimal costs associated with the initial investment of e.g., equipment and the equipment’s ongoing operation. Hence, the examined operation strategy has already been implemented at several (sludge-based) full-scale plants in Denmark. The energy needed to enhance the reactor temperature was balanced out by a minimum amount of extra biogas produced, making temperature increase energy efficient. The presented results provide important insight on the effect of small temperature differences in anaerobic microbial communities.
